# Double Q-Learning for Radiation Source Detection

**DOI:** 10.3390/s19040960

**Published:** 2019-02-24

**Authors:** Zheng Liu, Shiva Abbaszadeh

**Affiliations:** Department of Nuclear, Plasma, and Radiological Engineering, University of Illinois at Urbana-Champaign, 104 S Wright St, Urbana, IL 61801, USA; zliu86@illinois.edu

**Keywords:** reinforcement learning, radiation detection, source searching

## Abstract

Anomalous radiation source detection in urban environments is challenging due to the complex nature of background radiation. When a suspicious area is determined, a radiation survey is usually carried out to search for anomalous radiation sources. To locate the source with high accuracy and in a short time, different survey approaches have been studied such as scanning the area with fixed survey paths and data-driven approaches that update the survey path on the fly with newly acquired measurements. In this work, we propose reinforcement learning as a data-driven approach to conduct radiation detection tasks with no human intervention. A simulated radiation environment is constructed, and a convolutional neural network-based double Q-learning algorithm is built and tested for radiation source detection tasks. Simulation results show that the double Q-learning algorithm can reliably navigate the detector and reduce the searching time by at least 44% compared with traditional uniform search methods and gradient search methods.

## 1. Introduction

In the field of homeland security, detecting anomalous radiation sources in urban environments is an important yet challenging task due to the complexity of urban radiation background. When a suspicious area is determined, a radiation survey is usually carried out to search for anomalous radiation sources. To deliver a comprehensive and efficient survey, different survey approaches have been studied such as manually scanning of the area by human operated detectors and automatically scanning the area with robots under the navigation of pre-defined survey paths [1,2]. However, neither of the manual scanning method and the survey path method can achieve flexibility and efficiency at the same time. For instance, manual scanning carried out by humans is flexible to adjust survey strategies using new acquired measurements, but it requires lots of human efforts and may be dangerous for human surveyors. The pre-defined survey path method does not require human efforts during survey, but it cannot use new information gained during the survey and thus is not flexible to adjust survey paths. To address those issues, this study proposes the reinforcement learning algorithm as a data-driven approach to navigate automated robots equipped with radiation detectors for radiation source detection tasks.

Radiation exists everywhere in daily life. It arises from naturally occurring radioactive materials (NORMs) that are present in air, soil, and building materials [3,4]. Those NORMs contribute to the background radiation of the environment and are treated as a uniform radiation source. Besides those NORMs, there may also exist anomalous radiation sources in the environment such as wrongly disposed radioactive medical wastes, illicit radioactive materials, or even nuclear weapons [5,6]. They usually have high concentration with small footprint and are treated as point sources in the environment. The anomalous radiation source detection task is to identify and localize the anomalous radiation sources in an area. There are two major approaches that are commonly used to estimate the location and intensity of anomalous radiation sources from radiation measurements: the maximum likelihood estimation-based methods [7,8,9,10] and the Bayesian estimation-based methods [11,12,13]. In real applications, the radiation measurements are usually short in counts due to the limited detection time and the shielding of the radiation sources. The spectral comparison ratio method [14] and Gaussian process [15] have been studied to process the count-starved radiation measurements and suppress noise.

Besides those source parameter estimation studies, people also investigated into the optimized operations of detectors such as the placement of detectors and the optimized detection time. Klimenko et al., studied the problem of optimizing detection time for a predefined survey path using methods from the sequential testing theory [16]. Cortez et al., designed radiation surveys based on variances in acquired measurements and uncertainties in the radiation field [17]. Hutchinson et al., sequentially determined the detector’s placement positions using the concept of maximum entropy sampling [18]. Lazna et al., proposed a circular path planning strategy to exploit the directional characteristics of detectors [19]. Ristic et al., designed the survey path incrementally by choosing detector positions and detection times maximizing the information gain in the Renyi divergence sense [20]. Besides those conventional heuristic- or information theory-based methods, neural network-based reinforcement learning provides another emerging tool to solve the radiation source detection task.

Reinforcement learning (RL) is one of the machine learning algorithms that studies the problem of how agents ought to take actions in a given environment such that a certain goal can be achieved or rewards can be maximized [21]. With the recent development of computation hardwares and deep neural networks (DNN), the combination of RL and DNN shows great success in handling complex tasks such as playing video games, driving vehicles, playing Go, and controlling robots, to name a few [22]. By properly defining the learning problem, agents are able to learn the optimal actions under complex environments without prior human knowledge. Q-learning is one of the RL algorithms that learns the optimal action policy for agents without requiring a model of the environment [23]. Mnih et al., combined DNN with Q-learning which greatly improved the representability of Q-learning approach [24]; in their paper, the deep Q-learning (DQN) algorithm was trained to play Atari games and achieved human-level performance. Furthermore, the double Q-learning algorithm was developed to solve DQN’s issue of overestimating action values and achieved a more stable and reliable learning [25]. The above implement of Q-learning approaches show the potential to apply Q-learning to automated anomalous source detection tasks.

The major contribution of this paper is formulating the radiation source detection task as a reinforcement learning problem and constructing a double Q-learning algorithm to solve the radiation source detection task. In this study, a simulation environment is setup that an agent carrying a radiation detector (this agent with detector is called “the detector” in the remaining part of this paper for brevity) searches a predefined area. This area has background radiation and an anomalous radiation source. The goal of the detector is to find the source using as short time as possible. A convolutional neural network-based double Q-learning algorithm is built to navigate the detector looking for the radiation source. This paper is organized as follows: section two introduces the radiation source detection task, the simulation environment, and the double Q-learning algorithm for radiation source detection; section three presents training results of the algorithm and analyzes its performance in searching for radiation sources; section four summarizes this paper and discusses possible future steps.

## 2. Materials and Methods

### 2.1. Radiation Source Detection Task

According to the physics of radiation emission, radiation counts measured by radiation detectors during a unit time interval can be modeled by Poisson distribution:

(1) $$P\left(k;\lambda \right)=\frac{e^{-\lambda }\lambda^{k}}{k!}$$

Here λ is the intensity of the radiation, and *k* is the radiation counts measured by the detector. λ is contributed by two sources: the background radiation with intensity *b* and the anomalous radiation source with intensity *I*.

(2) $$\lambda =b+\frac{I}{d^{2}}*{𝟙}_{\left\{not\;blocked\right\}}$$

**Figure 1:**
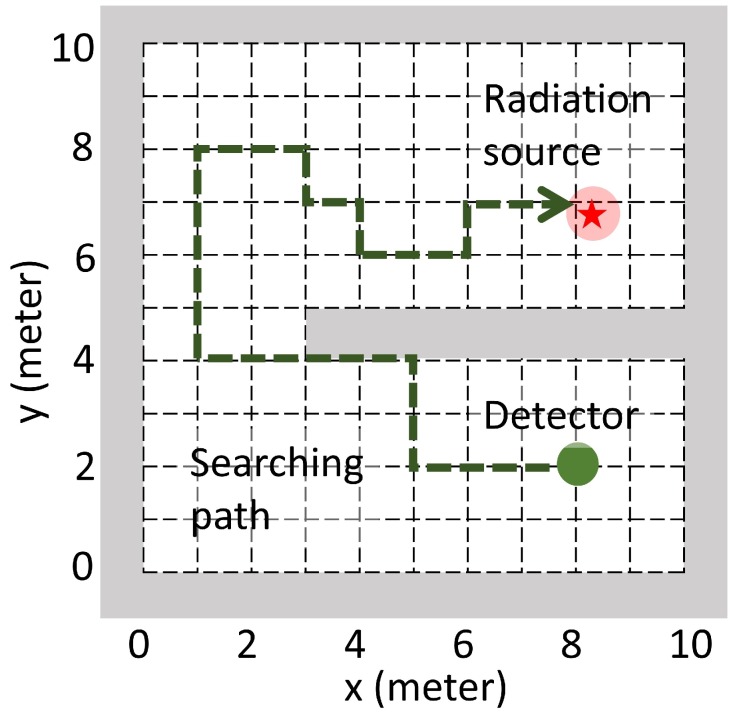
Illustration of the radiation detection task. The gray area denotes the searching area boundary and the walls inside the searching area. The green dashed line with arrow denotes a searching path of the detector.

The $d^{2}$ in Equation (2) is the distance between the detector and the radiation source. It is from the geometric efficiency correction that the detected point source’s radiation intensity is proportional to the point source’s solid angle viewed from the detector’s detection surface. This solid angle is further approximated to be squared inverse proportional to the distance (denoted by *d*) between the detector and the point radiation source. As shown in Figure 1, there may exist walls in the searching area that block the radiation source’s signal from the detector. Thus, there is an indicator function in Equation (2) to differentiate the blocked/not blocked case.

In this work, a radiation detection task and its simulation environment was setup according to the radiation model above. As shown in Figure 1, a searching area was setup in the simulation environment. In this area, there existed an anomalous radiation source and a radiation detector. In real life, radiation detectors usually move in all directions with arbitrary step size; in the simulation environment, the detector’s moving directions were limited to up, down, left, or right, and the step size was limited to 1 meter. Theses constrains simplified the movement control while kept a valid approximation to the real life scenario. With these constrains, the detector could only move along the dashed grids as shown in Figure 1. There might also have walls in the simulation environment that could totally block the source’s radiation signal. In the radiation detection task, the detector aimed at finding the anomalous radiation source as soon as possible.

### 2.2. Q-Learning with Convolutional Neural Network

In this work, we applied the double Q-learning approach [25] from reinforcement learning to train the detector searching for radiation sources. The radiation source detection task was formulated as a finite discrete Markov decision process (MDP), in which the radiation detector interacted with the environment through a sequence of states (*s*), actions (*a*), and rewards (*R*). The goal of the detector was to move to the grid node closest to the source as soon as possible. This was achieved by training a convolutional neural network (CNN) to approximate the optimal action value function $Q^{*}\left(s,a\right)$

(3) $$Q^{*}\left(s,a\right)=max_{\pi }E\left[\sum_{t^{\prime }=0}\gamma^{t^{\prime }}R_{t^{\prime }}|s,a,\pi \right],$$

which is the maximum expected cumulative future reward starting from state *s*, action *a*, discounted by γ, maximized over action policy π, and accumulated for future time steps $t^{\prime }$ (see Appendix A).

**Figure 2:**
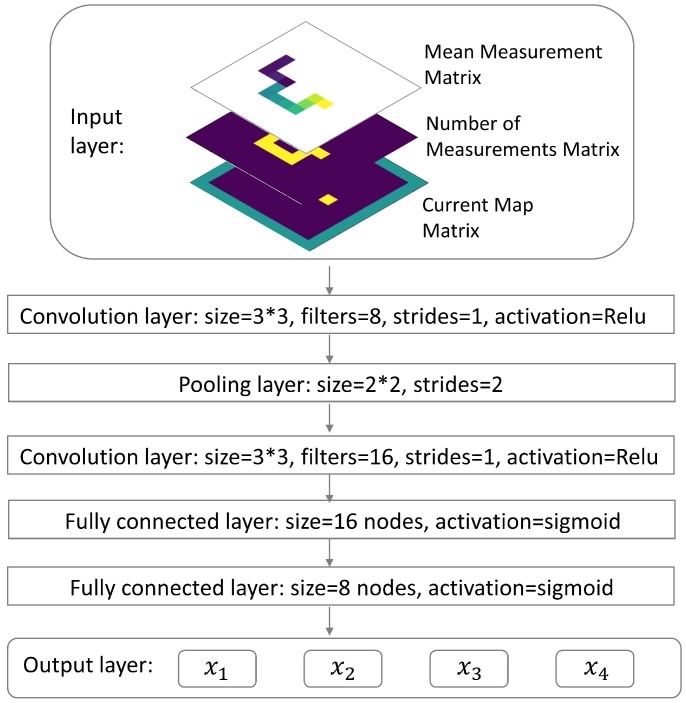
Schematic illustration of the convolutional neural network (CNN). The input of this CNN contains three images. The first image shows the mean radiation measurement for each of the visited positions. The second image shows the number of measurements for each position. The third image shows the detector’s current position in the searching area. The output layer is a fully connected layer with 4 nodes, and each of the nodes represents its corresponding action’s expected cumulative future reward given the input as current state. For the convolution layers or the pooling layer, the ’size’ parameter specifies the size of the convolution or pooling kernel; the ’filters’ parameter specifies the channel number of the convolution kernel; the ’strides’ parameter specifies the stride of the sliding window for each dimension of input; the ’activation’ parameter specifies the activation function applied to the output of the convolution results. For the pooling layer, we applied max pooling. For the fully connected layers, the ’size’ parameter specifies the number of nodes in the layer, and the ’activation’ parameter specifies the activation function applied to the output of the fully connected layer. The ’Relu’ activation function is defined as Relu(x)=max(x,0), and the ’Sigmoid’ activation function is defined as $Sigmoid\left(x\right)=\frac{e^{x}}{1+e^{x}}$. These definitions and names are consistent with the names used by the ’tf.layers.conv2d’ function in Tensorflow [26].

As shown in Figure 2, the CNN takes the state $s_{t}$ as input and outputs four different values $\left(x_{1},x_{2},x_{3},x_{4}\right)$ corresponding to four different actions ($a_{1}$=up, $a_{2}$=down, $a_{3}$=left, $a_{4}$=right). Each value represents the expected cumulative future reward starting from state $s_{t}$ and taking the corresponding action, for example $x_{i}=Q\left(s_{t},a_{i}\right),\forall i\in \left\{1,2,3,4\right\}$. Since the detector could only move along dashed grids (Figure 1), the state $s_{t}$ was constructed by several matrices, which preserved the detector’s searching history.

**Figure 3:**
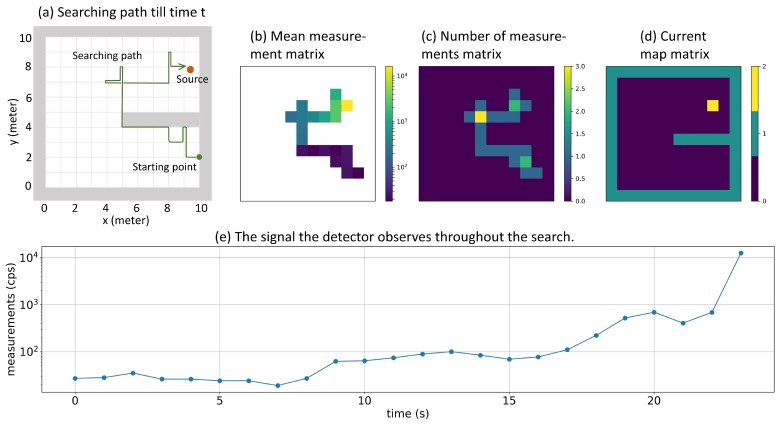
Representing the searching history using Mean measurement matrix, Number of measurements matrix, and Current map matrix. Plot (**a**) shows an example of a searching history. In plot (**b**), color represents the intensity of mean radiation measurements for each position. In plot (**c**), color represents the number of measurements been taken so far at each position. In plot (**d**), yellow represents the detector’s current position, green represents boundaries and walls, and blue represents accessible positions. Plot (**e**) represents the signal that the detector observed throughout the search.

If the simulation area is m meters wide and n meters long, a Mean Measurement Matrix of size m×n can be created so that the (i,j) element of this matrix represents the mean value of radiation measurements acquired at position (i,j). Similarly, a Number of Measurements Matrix of size m×n can be created so that the (i,j) element of this matrix stores how many measurements have been taken at position (i,j). A Current Map Matrix can also be created to record the position of the detector. Being different from previous two matrices, the Current Map Matrix has size (m+2)×(n+2) to incorporate boundaries of the simulation area. In this matrix, all boundaries and walls are denoted by 1, all accessible positions are denoted by 0, and the current position of the detector was denoted by 2. As shown in Figure 3, at each time step *t* these three matrices can be represented by three images. In our algorithm, the state $s_{t}$ was constructed as a stacking of these three images: the Mean Measurement Matrix and the Number of Measurements Matrix were first padded by zeros to let them have size (m+2)×(n+2), and then these three images were stacked together.

In our implementation, the simulated environment had an area of 10 m by 10 m, and we considered a typical hand-hold scintillation detector (a thallium activated cesium iodide detector with scintillator size 2×1×0.5 inch). The average background radiation level of the UIUC campus measured by this detector was 25 cps (counts per second); thus, *b* was set to be 25 cps in Equation (2). Plot (e) of Figure 3 shows an example of the signal the detector observed in one search. When training the reinforcement learning algorithm, the radiation source should be strong enough so that the detector can learn how to search for the source. Thus, *I* in Equation (2) was uniformly sampled from (3000, 7000) cps to simulate different anomalous radiation sources with different intensities. The double Q-learning algorithm was trained by iteratively conducting a large number of episodes, in which the detector interacted with the environment until it reached a certain terminal state, and the environment was reset for the next episode. In an episode, the simulation environment was randomly initialized so that the wall’s position, the radiation source’s position and intensity, and the detector’s position were properly defined. In each time step inside the episode, the radiation detector chose one direction to move and then collected one second radiation measurement. This episode would terminate if the detector moves to the grid node closest to the source, or the total number of time steps is larger than a predefined time limit. In this study, the time limit was chosen to be 100 because it is the time needed to traverse the entire simulation area. The double Q-learning algorithm was trained for 1 million episodes. The reward in each time step was defined as follows:

(4) $$R_{t}=\left\{\begin{array}{cc} 0.5, & \mathrm{if}\;\mathrm{the}\;\mathrm{detector}\;\mathrm{moves}\;\mathrm{closer}\;\mathrm{to}\;\mathrm{the}\;\mathrm{source}.\\ -1.5, & \mathrm{otherwise}. \end{array}\right.$$

Since there might exist walls, the Euclidean distance could not be directly used; the shortest-path distance was instead used to determine whether the detector moved closer or further to the source. This reward was set to be asymmetrical (0.5 vs. −1.5) to encourage the detector finding the source as soon as possible. The detailed training configuration is presented in Appendix B, and code is available upon request.

### 2.3. Evaluation

Three simulation experiments were carried out to evaluate the performance of the Q-learning algorithm. The first experiment was the radiation source searching test (Section 3.2), the second experiment was the detector trapping test (Section 3.3), and the third experiment was the radiation source estimation test (Section 3.4).

**Figure 4:**
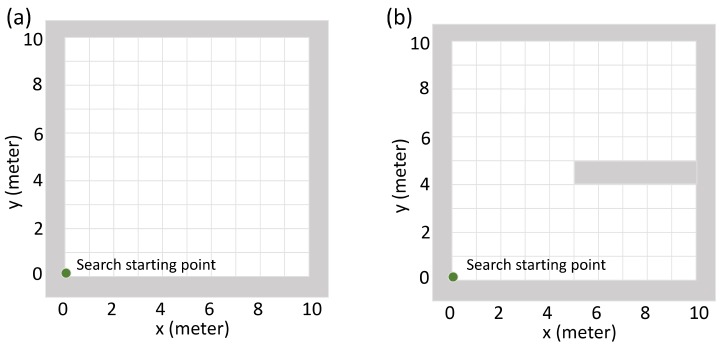
Illustration of the searching areas in the first experiment. (**a**) The searching area that does not have walls inside. (**b**) The searching area that has one wall inside. In both of the searching areas, the detector always starts searching from the lower left corner.

In the first experiment, the goal of the detector was to find the source as soon as possible. A successful case of finding a source was defined as the detector moving to the grid node closest to the source within 100 steps. For example, when source is at (5.2,7.8), the search is successful if the detector moves to the grid (5,8) within 100 steps. The metrics were the average searching time and the failure rate of finding the source. In this experiment, two different searching areas were tested as shown in Figure 4. One searching area does not have walls inside, and the other searching area has one wall inside. For both of the searching areas, the detector was always initialized at the lower left corner of the searching area. In each of the searching areas, we tested ten different source intensities from $50\times 2^{0}\;cps$ to $50\times 2^{9}\;cps$. For each source intensity, we repeated the search 200 times with different random source positions. The performance of the Q-learning algorithm was compared with the gradient search algorithm (Appendix C).

**Figure 5:**
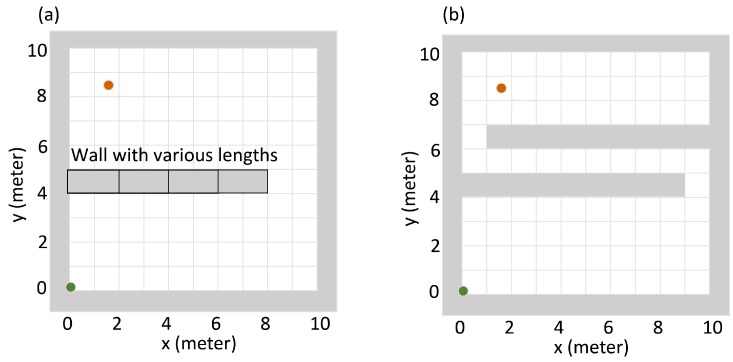
Illustration of the simulation areas in the detector trapping test. Green dots represent the initial point of the detector, and red dots represent the position of the radiation source. Grey areas represent the walls which can block the source’s radiation entirely. Plot (**a**) shows the first kind of simulation area, in which the wall is attached to the left edge of the simulation area and has different lengths (0 m, 2 m, 4 m, 6 m, 8 m). The longer the wall is, the harder it is for the detector to move from lower part to the upper part. All these wall configurations have been seen by the Q-learning algorithm in the training stage. Plot (**b**) shows the second kind of the simulation area. This wall configuration has not been seen by the Q-learning algorithm in the training stage.

In the second experiment, we compared the detector trapping behavior between the Q-learning algorithm and the gradient search algorithm. As shown in Figure 5, there were two different kinds of simulation areas to be tested. In both of the areas, the detector started from the lower left corner, and the source was placed at the upper left corner. In order to find the source, the detector needed to move from the lower part to the upper part of the simulation area. The plot (a) in Figure 5 shows the first kind of simulation area, in which the wall was attached to the left edge of the area and had various lengths (0 m, 2 m, 4 m, 6 m, 8 m). The longer the wall is, the more radiation signal it will block. All these wall configurations were already seen by the Q-learning algorithm in the training stage. The plot (b) in Figure 5 shows the second kind of simulation area. This wall configuration was not seen by the Q-learning algorithm in the training stage. For each of the wall configurations, 20 repeated tests were performed. For each test, we computed the relative trapped time, which was defined as the percentage of time the detector stayed in the lower part of the simulation area (y∈[0,4]) during the whole searching process. The larger the relative trapped time is, the severer the detector is trapped.

In the third experiment, the goal of the detector was to estimate the accurate location and intensity of the radiation source. Similar to the first experiment, the detector was guided by a navigation algorithm to look for the radiation source. The navigation algorithms under comparison were the Q learning algorithm, the gradient search algorithm, and the uniform search algorithm [27] with three different searching densities (see Appendix C). After the search, we estimated the radiation source’s location and intensity using measurements collected so far. The closer the detector is to the source, the more informative radiation measurements can be acquired by the detector, and consequently a more accurate estimation about the source can be made. There are a variety of algorithms to estimate the radiation source’s location and intensity [7,8,9,10,11,12,13]. For simplicity, we used the maximum likelihood estimation (MLE) approach to do the estimation. Suppose $\left\{\left(x_{1},y_{1},m_{1}\right),\left(x_{2},y_{2},m_{2}\right),\cdots ,\left(x_{t},y_{t},m_{t}\right)\right\}$ are the measurements acquired till time *t*, the MLE estimator of source position $\left(x_{s}^{*},y_{s}^{*}\right)$ and source intensity $\mu_{s}^{*}$ is calculated as follows:

(5) $$\begin{array}{c} \left(x_{s}^{*},y_{s}^{*},\mu_{s}^{*}\right)=argmax_{\left(x_{s},y_{s},\mu_{s}\right)}\sum_{i=1}^{t}\left(m_{i}\times log\left(\lambda_{i}\right)-\lambda_{i}\right), \end{array}$$

(6) $$\begin{array}{c} \lambda_{i}=b+\frac{\mu_{s}}{{\left(x_{i}-x_{s}\right)}^{2}+{\left(y_{i}-y_{s}\right)}^{2}}*{𝟙}_{\left\{not\;blocked\right\}}. \end{array}$$

**Figure 6:**
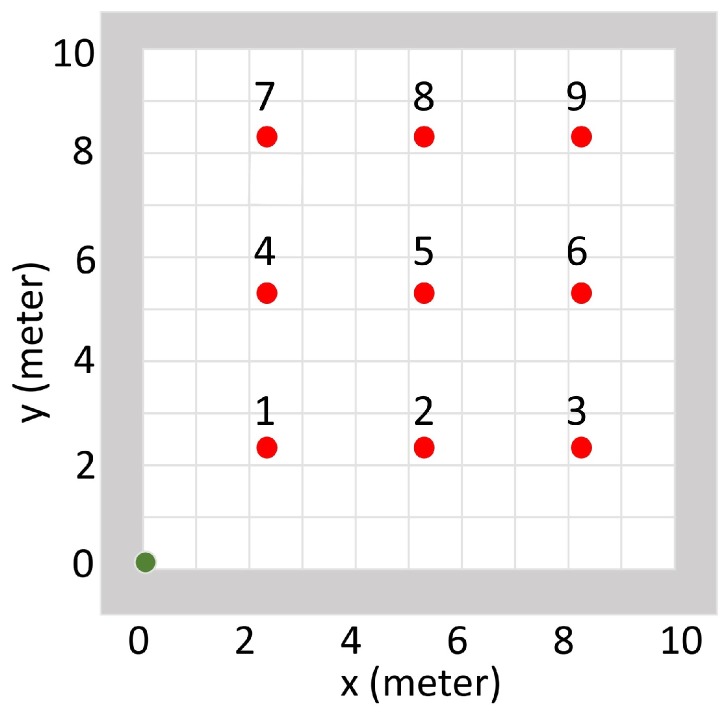
Experiment setup for the radiation source estimation test. The simulation area does not have walls inside. The green dot illustrates the starting point of the detector, and the red dots illustrate nine different testing source positions indexed from 1 to 9.

In this experiment, the simulation area did not have walls inside, and nine different source positions were tested (Figure 6). The source intensity was set to be 500 cps. For each source position, 20 repeated tests were performed.

## 3. Results and Discussion

### 3.1. Training Result of the Double Q-Learning Algorithm

**Figure 7:**
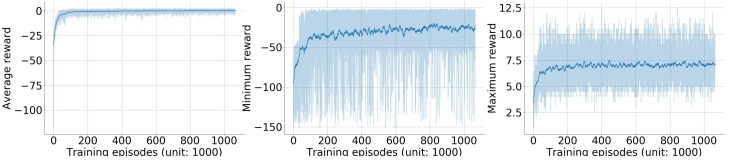
Training curves of the episode reward. One episode is one round of the searching task, starting from initializing the simulation environment and ending with the detector triggering the termination condition (either finding the source or reaching the maximum time step inside an episode). Between every 100 training episodes, 30 randomly initialized episodes were evaluated with CNN parameters fixed. Plot (**a**) shows the average testing reward of the 30 episodes. Plot (**b**) shows the minimum testing reward of the 30 episodes. Plot (**c**) shows the maximum testing reward of the 30 episodes. In these three plots, dark blue lines represent the averaged testing results with window size of 100 episodes, while light blue shadows represent raw testing results.

The training progress of the double Q-learning algorithm was monitored through a series of checkpoints. Between every 100 training episodes there was a checkpoint. In each checkpoint, 30 randomly initialized episodes were performed with CNN parameters fixed, and the average, minimum, and maximum reward in these episodes were recorded (Figure 7).

The average reward converged to near zero after 400 k episodes of training. Note that the per-step reward function was asymmetric (Equation (4)), an episode reward with zero mean meant that for every three correct actions (move closer to the source) the detector would take one wrong action (move further away from the source) on average. The minimum reward had majority between −50 and 0 with mean value converging to −25. It delivered a much bigger fluctuation than the mean reward. This fluctuation was caused by rear scenarios that were not learned well by the algorithm. The maximum reward converged to 7 with much smaller fluctuation than the minimum reward.

### 3.2. Radiation Source Searching Test

To further analyze the Q-learning algorithm’s performance in radiation source searching tasks, we selected the Q-learning model learned at the 842,500th episode and tested its performance under two different searching areas as shown in Figure 4. This model was selected because it achieved the biggest minimum reward during the training process (the middle plot of Figure 7). Metrics under evaluation were the average searching time and the failure rate of finding a radiation source. The Q-learning model was also compared with the gradient search approach.

**Figure 8:**
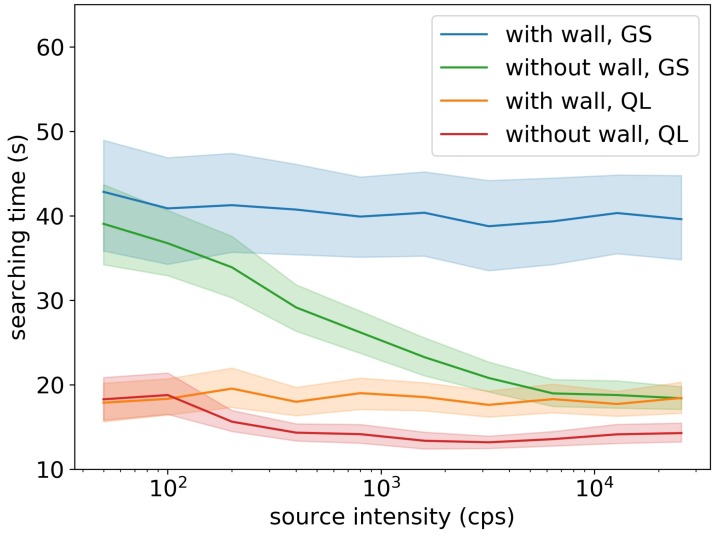
Average searching time under different source intensities. The shaded area is the 95% confidence interval estimated by bootstrapping. The ’GS’ stands for the gradient search method, and the ’QL’ stands for the Q-learning method. The Q-learning method used less searching times than the gradient search method in all simulation conditions.

As shown in Figure 8, the Q-learning algorithm outperformed the gradient search method in both simulation areas with walls and without walls, in all tested source intensities. Because walls could block radiation and introduce obstacles in detector’s movements, adding walls made the searching time longer for both of the two algorithms. For simulation areas without walls, two algorithms’ searching times reduced when the source intensity increased, and the Q-learning algorithm spent at least 25% less searching time than the gradient search method. For simulation areas with walls, the two algorithm’s searching times did not depend on the source intensity, and the searching time of the Q-learning algorithm was 50% less than the searching time of the gradient search method.

**Figure 9:**
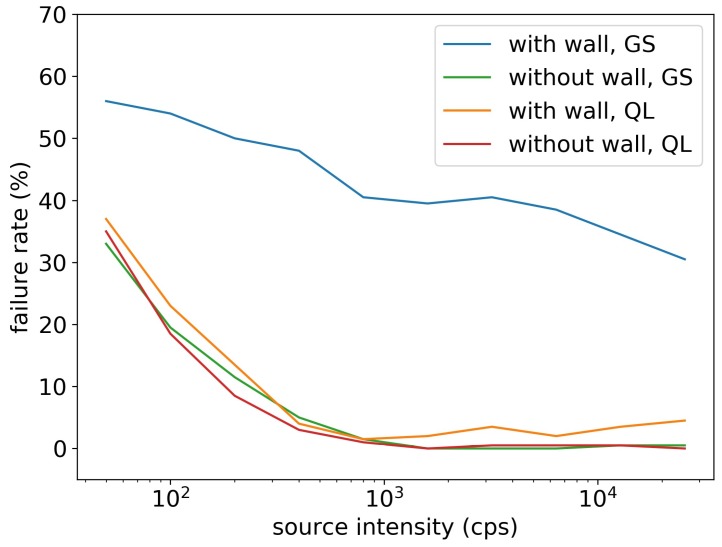
Failure rate of the Q-learning algorithm (QL) and the gradient search algorithm (GS) in the radiation source searching test, for simulation areas with walls and without walls. Without walls, the two algorithms had the same failure rate; with walls, the Q-learning algorithm achieved much smaller failure rate than the gradient search method.

Figure 9 shows that the failure rate of the gradient search algorithm and the Q-learning algorithm significantly dropped when the source intensity increased. Without walls, the failure rate of the two algorithms were the same and converged to below 2% for sources stronger than 800 cps. With walls, the failure rate of the Q-learning algorithm slightly increased and converged to 5% for sources stronger than 800 cps, but the gradient search method’s failure rates were all above 30%. The gradient search method relied solely on radiation gradient to search for sources. If the detector and the source were in different sides of the wall, there would be no radiation gradient in the detector side, and the detector would just randomly move until it moved to the other side of the wall. This behavior significantly reduced the performance of the gradient search method, especially when the searching area had obstacles. On the contrary, the Q-learning method was able to design its optimal searching path based on the obstacles in the searching area. If the detector found the source did not exist in this side of the wall, it would take the shortest path to the other side of the wall and search for sources. This explained why the Q-learning algorithm performed equally well in areas with walls and without walls.

### 3.3. Detector Trapping Test

The previous experiment reveals the Q-learning algorithm’s ability to avoid being trapped by the walls. In this experiment, we further analyzed the detector trapping issue of the Q-learning algorithm and the gradient search algorithm on two different simulation areas shown in Figure 5.

**Figure 10:**
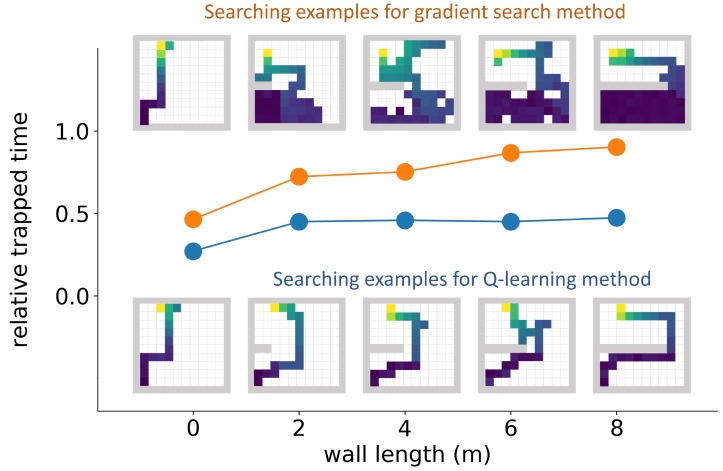
Detector trapping test 1. In this trapping test, simulation areas with different wall lengths were tested. The relative trapped time was defined as the percentage of the time the detector stayed in the lower 40% part of the simulation area during the whole searching process. The orange curve represents the mean relative trapped time of the gradient search method, and the blue curve represents the mean relative trapped time of the Q-learning method. The example searching paths for both algorithms in different wall lengths are also drawn in the figure. For all the searching path examples, the detector initialization point was at the lower left corner, the radiation source was at the upper left corner, and the color of the path represents the mean radiation intensity (blue is low, and yellow is high). The Q-learning algorithm was not trapped by walls, while the gradient search method was trapped by walls.

Figure 10 shows the average relative trapped times (curves) and searching path examples (drawings) for the Q-learning algorithm and the gradient search algorithm in simulation areas illustrated by plot (a) of Figure 5. The Q-learning model under evaluation was taken from the 842,500th episode. As the wall length increased from 0 m to 8 m, the relative trapped time of the gradient search method increased from 0.5 to 0.9. From the searching path examples we can find that the gradient search method was easily trapped by long walls and wasted most of the searching time in the wrong side of the wall. When there was no wall, the Q-learning algorithm’s relative trapped time was 0.27. When there were walls, its relative trapped times increased to 0.46 and were independent of the wall length. This demonstrates that the Q-learning algorithm was not trapped by walls. Example searching paths from the Q-learning algorithm show efficient searching strategies.

**Figure 11:**
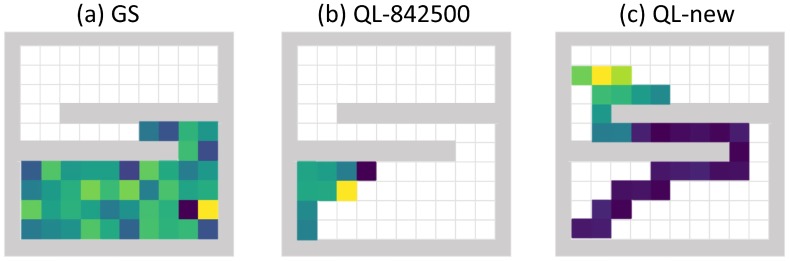
Detector trapping test 2. In this trapping test, a simulation area with two overlapped long walls was tested. The relative trapped time was defined as the percentage of the time the detector stayed in the lower 60% part of the simulation area during the whole searching process. For all the searching path examples, the detector initialization point was at the lower left corner, the radiation source was at the upper left corner, and the color of the path represents the mean radiation intensity (blue is low, and yellow is high). Plot (**a**) shows an example search path from the gradient search method that was failed to find the source. Plot (**b**) shows an example search path from the Q-learning model taken from the 842,500th episode, and this search was also failed to find the source. Plot (**c**) shows an example search path from the Q-learning model that was trained for additional 6000 episodes on the new simulation area. After this additional training, the new Q-learning model was able to efficiently search for sources in the new geometry.

In the previous detector trapping test, one possible reason for the Q-learning algorithm not being trapped by walls is that the tested simulation areas are similar to the ones that have already been seen by the Q-learning algorithm during the training stage. In order to evaluate the detector trapping performance in new simulation areas, we tested the Q-learning algorithm and the gradient search method in the simulation area illustrated by plot (b) of Figure 5. This simulation area has different geometry compared to the ones being used to train the Q-learning algorithm. The overlapped two walls blocked all the source’s radiation from the initialization point of the detector. In this test, the relative trapped time was defined as the percentage of the time the detector stayed in the lower 60% of the searching area. The gradient search algorithm’s mean relative trapped time was 0.999, and plot (a) of Figure 11 shows a failure search path. For this geometry, the detector in the lower part of the simulation area could not receive any signal from the radiation source in the upper part of the simulation area, and the gradient method would just randomly pick one direction to move since it could not detect any meaningful radiation gradient. The gradient search algorithm was heavily trapped by this geometry, and almost all of its searches failed. The Q-learning model taken from the 842,500th episode had a mean relative trapped time of 1, which means that this Q-learning model was also entirely trapped by the new geometry. Plot (b) of Figure 11 shows a failure search path of this Q-learning model. We can see that this detector was trapped in the corner and did not search for the source at all. It is because the trained Q-learning algorithm was highly fitted to the training simulation areas and did not generalize well for the simulation area with new geometry. To teach the Q-learning algorithm how to search in this new geometry, we added this new geometry into the training stage and trained 6000 additional episodes starting from the 842,500-episode model. After this additional training, the new Q-learning algorithm was not trapped by walls any more and achieved a relative trapping time of 0.63. Plot (c) of Figure 11 shows a successful search path from the new Q-learning algorithm. In this search, the algorithm firstly realized that there was no source in the lower part of the area; then, it took the shortest path to the upper part of the area and finally found the source there.

This experiment demonstrates that the Q-learning algorithm is able to efficiently design its searching path based on searching area geometries and recent radiation measurements. For learned search area geometries, the Q-learning algorithm will not be trapped and can find the source efficiently; for new search area geometries, the Q-learning algorithm will fail. This issue can be solved by adding additional training episodes for the new search area geometries. In our experiment, the 6000 additional training episodes took less than 30 min on a Tesla K80 GPU. In real applications, the searching area geometry is usually available in advance (such as through satellite pictures from Google Maps), and the Q-learning model can be easily updated according to the target searching area geometry in short time.

### 3.4. Radiation Source Estimation Test

In this experiment, we tested the Q-learning algorithm, the uniform search algorithm, and the gradient search algorithm’s performance of estimating the source’s position and intensity. Similar to the previous section, the Q-learning model under evaluation was taken from the 842,500th episode.

Table 1 shows the mean estimation error for different searching methods. The simulation area was 10 m by 10 m. All of the five searching methods achieved source localization errors less than 15 cm (relative errors less than 1.5%) and source intensity estimation errors less than 7%. Averaging over all the 9 source positions shown in Figure 6, the mean searching time was 15.82 s for the Q-learning algorithm and 33.63 s for the gradient search algorithm. The Q-learning approach was 50% quicker than the gradient search algorithm and at least 44% quicker than the uniform search algorithms. Compared to the gradient search method, the Q-learning algorithm reduced the localization error by 35%, obtained similar source intensity estimation error, and used less searching time. Compared to the uniform search 1 approach, the Q-learning approach used less detection time and achieved a smaller localization error. The trade-off was a slightly bigger intensity estimation error (6.3% vs. 5%). The uniform search 2 and 3 approaches outperformed the Q-learning method, but they used much longer searching time and collected much more measurements. For source searching tasks with limited searching time, the Q-learning method would be a competitive alternative method compared to the uniform search method and the gradient search method, since it significantly reduced the searching time while maintained a low estimation error.

## 4. Conclusions and Future Works

In this paper, the radiation source detection task was formulated as a reinforcement learning problem and a CNN-based double Q-learning algorithm was developed to navigate the detector searching for the radiation source. Simulation environments were set up to test the algorithm’s performance. In the radiation source searching test, the Q-learning method used at least 25% less searching time and achieved a lower failure rate than the gradient search algorithm. In the detector trapping test, the Q-learning algorithm was less prone to the detector trapping issue than the gradient search method. In the radiation source estimation test, all of the uniform search methods, the gradient search method, and the Q-learning method achieved relative localization error lower than 1.5% and relative intensity estimation error lower than 7%, but the Q-learning approach reduced the mean searching time by at least 44% compared to other methods.

In the future, we are going to implement this Q-learning algorithm on a drone-based radiation detection platform, and field tests will be carried out. In real applications such as nuclear security and nuclear decommissioning, the detection platform’s computation power and energy supply are usually limited. For security concerns, the detection platform may need to be isolated from Internet connection. These limitations require the navigation algorithm to run locally on light-weight mobile computation devices such as mobile phones. The Q-learning algorithm’s computation burden is mostly from the training stage, which could be done in other powerful computers. Once the training is finished, the algorithm can be deployed efficiently on light-weight mobile devices for navigation.

The current implementation of the double Q-learning algorithm has fixed detection time (1 s for each time step), fixed step length (1 m for each action), and limited moving angles (4 directions). In the future, we will explore the finer controlling of the detector such as adding detection time as another controllable parameter, accepting different step lengths, and supporting more moving angles. The current Q-learning algorithm was designed and trained based on the one-source searching scenario; consequently, it could only handle one-source searching tasks. However, it has the potential to be applied into multiple-source searching tasks. When more than one source present in the environment, the current algorithm can still move towards one of the sources, but it does not know what to do after finding the first source. In order to search for multiple sources, the Q-learning algorithm needs to remember the found sources and use their positions and intensities to adjust new radiation measurements.

## Figures and Tables

**Table 1:** Radiation source estimation test.

Approach *	Mean Estimation Error			Mean Searching Time (s) **								
	$x_{s}\left(m\right)$	$y_{s}\left(m\right)$	$\mu_{s}\left(\%\right)$	$t_{1}$	$t_{2}$	$t_{3}$	$t_{4}$	$t_{5}$	$t_{6}$	$t_{7}$	$t_{8}$	$t_{9}$
QL	0.057	0.064	6.3	7.85	16.70	22.35	6.90	15.70	17.75	15.65	16.75	22.70
GS	0.090	0.101	6.5	11.2	17.2	31.3	17.85	21.15	52.1	38.8	37.0	76.05
U1	0.082	0.134	5.0	28								
U2	0.024	0.026	3.1	37								
U3	0.013	0.011	1.7	54								

* QL: Q-learning; GS: gradient search; U1: uniform search 1; U2: uniform search 2; U3: uniform search 3 (Appendix C.2); ** $t_{i}\left(i\in \left[1,9\right]\right)$ represents the mean searching time for the i’th source shown in Figure 6.

**Figure A1:**
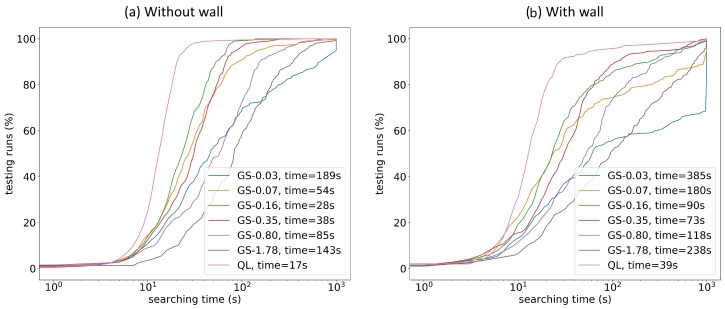
Parameter tuning for the gradient search method. These two plots represent the percentage of testing runs that are finished within specific searching times. Plot (**a**) shows the results from simulation area without walls, and plot (**b**) shows the results from simulation area with walls. ’GS’ stands for the gradient search method, the number after ’GS’ represents the *q* value. ’time’ represents the mean searching time for that specific setting. ’QL’ stands for the Q-learning method. Without walls, the best *q* is 0.16; with walls, the best *q* is 0.35.

**Figure A2:**
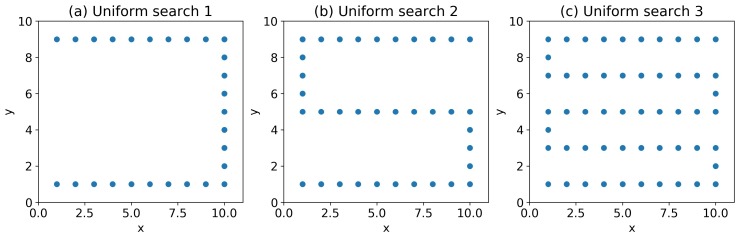
The uniform search algorithms with different searching densities. Each blue dot represents a position to take one measurement. From (**a**) to (**c**), their total search times are 28 s, 37 s, and 54 s respectively.

## Appendix A. Q-Learning and Radiation Source Detection

In this section, the double Q-learning algorithm for radiation source detection task is described in details. We firstly describe the radiation source detection model, then introduce the Bellman equation in Q-learning, and finally explain how a convolution neural network (CNN) is constructed and trained according to the Bellman equation.

### Appendix A.1. Radiation Source Detection Model

The setup of radiation source detection task is described in Section 2.1: the detector aims to find the source within the given search-time limit *T*. Because we also assume that the detector will move one step in each second, this search-time limit *T* is also the upper limit for the number of steps the detector can take to look for the source. At time *t* (0≤t≤T), the detector acquires one radiation measurement $k_{t}$ at position $\left(x_{t},y_{t}\right)$, takes one action $a_{t}$, and receives one reward $R_{t}$. The measurement $k_{t}$ is sampled based on the Poisson model being described in Equations (1) and (2), the action $a_{t}$ is chosen from the action set {move up, move down, move left, move right}, and the step reward $R_{t}$ is determined by Equation (4). The state of the detector at time *t* is denoted by $s_{t}$, which is constructed by all the historical measurements and their positions up to time *t*: $s_{t}=f\left(\left(x_{0},y_{0},k_{0}\right),\cdots ,\left(x_{t},y_{t},k_{t}\right)\right)$. In this paper, we construct the state $s_{t}$ as a collection of three different matrices: the mean measurements matrix, the number of measurements matrix, and the current map matrix. Those matrices are represented as figures in the input layer of the CNN shown in Figure 2. With this construction, we can easily verify the Markov property of the states:

(A1) $$P\left(s_{t+1}|a_{t},s_{t},s_{t-1},\cdots ,s_{0}\right)=P\left(s_{t+1}|a_{t},s_{t}\right)$$

### Appendix A.2. Bellman Equation in Q-Learning

In this paper, the Q-learning algorithm aims to find the radiation source as soon as possible. This is achieved by learning an action strategy to maximize a cumulative future reward:

(A2) $$G_{t}=\sum_{t^{\prime }=0}^{T-t}\gamma^{t^{\prime }}R_{t+t^{\prime }}.$$

γ is a discounting factor between zero and one that reduces the importance of far-future rewards, and $G_{t}$ is the cumulative future reward starting at time *t*. In the radiation source detection task, the detector chooses its action $a_{t}$ according to a probability-based policy π. $\pi (a_{t}|s_{t})$ specifies the probability of the detector to take action $a_{t}$ when it observes state $s_{t}$. With a certain policy π, the action-value function $Q_{\pi }\left(s,a\right)$, also known as Q-function, describes the expected cumulative future reward starting from state *s*, action *a*, and using policy π:

(A3) $$Q_{\pi }\left(s,a\right)=E\left[G_{t}|S_{t}=s,A_{t}=a,\pi \right]$$

The optimal action-value function $Q^{*}\left(s,a\right)$ is obtained by applying an optimal policy so that the action-value function $Q_{\pi }\left(s,a\right)$ is maximized:

(A4) $$Q_{\pi }^{*}\left(s,a\right)=max_{\pi }E\left[G_{t}|S_{t}=s,A_{t}=a,\pi \right].$$

$Q^{*}\left(s,a\right)$ represents the expected cumulative future reward starting from state *s* and action *a*, and using an optimal policy. Bring Equation (A2) into Equation (A4), we will get

(A5) $$\begin{array}{c} \begin{array}{c} \;Q^{*}\left(s,a\right)\\ =max_{\pi }\;E_{\left\{\left(S_{t+1},A_{t+1}\right),\left(S_{t+2},A_{t+2}\right),\cdots \right\}}\left[R_{t}+\gamma G_{t+1}|S_{t}=s,A_{t}=a,\pi \right] \end{array} \end{array}$$

(A6) $$\begin{array}{c} \begin{array}{c} =E_{S_{t+1}}\left[R_{t}|S_{t}=s,A_{t}=a\right]\\ \;+\gamma max_{\pi }E_{\left(S_{t+1},A_{t+1}\right)}\left[\;E_{\left\{\left(S_{t+2},A_{t+2}\right),\cdots \right\}}\left[G_{t+1}|S_{t+1},A_{t+1},\pi \right]\;|S_{t}=s,A_{t}=a,\pi \right] \end{array} \end{array}$$

(A7) $$\begin{array}{c} \begin{array}{c} =E_{S_{t+1}}\left[R_{t}|S_{t}=s,A_{t}=a\right]\\ \;+\gamma \;max_{\pi }\;E_{\left(S_{t+1},A_{t+1}\right)}\left[\;Q^{*}\left(S_{t+1},A_{t+1}\right)\;|S_{t}=s,A_{t}=a,\pi \right] \end{array} \end{array}$$

(A8) $$\begin{array}{c} \begin{array}{c} =E_{S_{t+1}}\left[R_{t}|S_{t}=s,A_{t}=a\right]\\ \;+\gamma \;E_{S_{t+1}}\left[\;max_{a^{\prime }}\;Q^{*}\left(S_{t+1},a^{\prime }\right)\;|S_{t}=s,A_{t}=a\right] \end{array} \end{array}$$

Finally, we obtain the Bellman equation [28] from the last line of above equation:

(A9) $$Q^{*}\left(s,a\right)=E_{S_{t+1}}\left[R_{t}+\gamma \;max_{a^{\prime }}Q^{*}\left(S_{t+1},a^{\prime }\right)\;|S_{t}=s,A_{t}=a\right]$$

The intuition of the Bellman equation is that the optimal policy will always choose the action that can maximize the expected cumulative future reward for the next time step. According to this Bellman equation, Watkins and Dayan proposed the following iterative updating algorithm known as Q-learning and proved its convergence [23]:

(A10) $$Q\left(S_{t},A_{t}\right)\leftarrow \left(1-\alpha \right)\;Q\left(S_{t},A_{t}\right)+\alpha \left[R_{t}+\gamma max_{a}Q\left(S_{t+1},a\right)-Q\left(S_{t},A_{t}\right)\right].$$

α is a parameter between 0 and 1 controlling the learning rate. The convergence of the above updating rule requires that all possible pairs of (a,s) are repeatedly visited during the training process.

### Appendix A.3. Neural Network for Q-Learning

Usually, interesting problems have a large number of states and actions, and thus the updating rule (Equation (A10)) is difficult to implement. Instead, a function approximator Q(s,a;θ) can be learned according to the Bellman equation. In this study, we use CNN as the function approximator, and θ represents the parameters (weights) of the CNN. In the Deep Q Network (DQN) paper [24], the target value is defined as

(A11) $$y=R_{t}+\gamma \;max_{a}Q\left(S_{t+1},a;\theta^{\prime }\right)$$

The $\theta^{\prime }$ is the parameter of the CNN from previous iterations and hold as constant. The loss function is defined according to the Bellman equation:

(A12) $$\begin{array}{cc} L(\theta ) & =E_{s,a}\left[\;{\left(Q\left(s,a;\theta \right)-E_{S_{t+1}}\left[y|S_{t}=s,A_{t}=a\right]\right)}^{2}\;\right] \end{array}$$

(A13) $$\begin{array}{c} =E_{s,a,S_{t+1}}\left[\;{\left(Q\left(s,a;\theta \right)-y\right)}^{2}\;\right]-E_{s,a}\left[Var_{S_{t+1}}\left(y|S_{t}=s,A_{t}=a\right)\right] \end{array}$$

The second term in Equation (A13) represents the variance of the target value which does not depend on θ and is thus ignored. The gradient of the estimation loss with respect to θ is

(A14) $$\nabla_{\theta }L\left(\theta \right)=E_{s,a,S_{t+1}}\left[\;\left(Q\left(s,a;\theta \right)-y\right)\nabla_{\theta }Q\left(s,a;\theta \right)\;\right]$$

At this point, θ can be learned using gradient descent approach:

(A15) $$\theta_{i+1}=\theta_{i}-\alpha \nabla_{\theta }L\left(\theta_{i}\right)$$

Experience replay [24] is used to calculate the expectation in the gradient (Equation (A14)) during the training process. In every training step, training data $\left(S_{t},A_{t},R_{t},S_{t+1}\right)$ are stored in a memory buffer. For each iteration, samples are uniformly selected from the memory buffer, and the CNN is updated through the batch stochastic gradient descent using those samples. This technique insures the independence of the training data between different training iterations and thus avoids oscillations or divergence in θ [24].

Double Q-learning [25] is used in this paper to further improve the performance. In DQN, Equation (A11) can be rewritten as following:

(A16) $$y=R_{t}+\gamma Q\left(S_{t+1},argmax_{a}Q\left(S_{t+1},a;\theta^{\prime }\right);\theta^{\prime }\right)$$

Because the action *a* is greedily selected from *Q* with parameter $\theta^{\prime }$ and then evaluated using *Q* with the same parameter $\theta^{\prime }$, it may lead to the overestimation of the action-value function. Double Q-learning approach addresses this issue by decoupling the selection from evaluation [25]:

(A17) $$y^{DoubleQ}=R_{t}+\gamma Q\left(S_{t+1},argmax_{a}Q\left(S_{t+1},a;\theta \right);\theta^{\prime }\right)$$

## Appendix B. Training Details

When training the model, we applied the ϵ-greedy [24] policy with ϵ reduced from 1 to 0.1 linearly over the first one million steps. During training, the loss calculated according to the mean squared-error (Equation (A12)) might be very big and have large gradients. This large gradients might make the training process unstable. To control the fluctuation of gradients and stabilize the training process, we replaced the original loss in Equation (A12) with the following Huber loss:

(A18) $$L\left(y,\hat{y}\right)=\left\{\begin{array}{cc} \frac{{\left(y-\hat{y}\right)}^{2}}{2}, & \mathrm{if}|y-\hat{y}|\leq 1.\\ |y-\hat{y}|-\frac{1}{2}, & \mathrm{otherwise}. \end{array}\right.$$

The double Q-learning algorithm used in this study is shown as follows:

**Algorithm A1** Double Q-learning with CNN.
1: Initialize reward function Q with weights θ 2: Initialize target reward function $\hat{Q}$ with weights $\hat{\theta }$ 3: Initialize replay memory D 4: **for** episode = 1,M **do** 5: Initialize states $\phi_{1}=\left(s_{1},n_{1},m_{1}\right)$ 6: **for** t = 1,T **do** 7: Select action $a_{t}$ using ϵ-greedy policy based on Q 8: Execute action $a_{t}$, obtain reward $r_{t}$ and state $s_{t+1}$ 9: Update states $\phi_{t+1}=\left(s_{t+1},n_{t+1},m_{t+1}\right)$ 10: Store transition $\left\{\phi_{t},a_{t},r_{t},\phi_{t+1}\right\}$ in D 11: Sample random minibatch of transitions $\left\{\phi_{j},a_{j},r_{j},\phi_{j+1}\right\}$ from D 12: Set $$y_{j}=\left\{\begin{array}{l} r_{j}\;\mathrm{if}\;\mathrm{episode}\;\mathrm{terminates}\;\mathrm{at}\;\mathrm{step}\;j+1\\ r_{j}+\gamma \hat{Q}\left(\phi_{j+1},\underset{a}{argmax}Q\left(\phi_{j+1},a\right)\right)\;\mathrm{otherwise} \end{array}\right.$$ 13: Perform a gradient descent step on HuberLoss$\left(y_{j},Q\left(\phi_{j},a_{j};\theta \right)\right)$ for θ 14: Every C steps reset $\hat{Q}=Q$ 15: Terminate the episode if source is found 16: **end for** 17: **end for**

$s_{t},n_{t}$, and $m_{t}$ represent the mean measurement matrix, number of measurements matrix, and current map matrix at step *t* respectively. The total number of training episodes was $M=1\times 10^{6}$, the step limit for each episode was T=100, the discount factor was γ=0.9, and the $\theta^{\prime }$ was updated every 10 training steps.

## Appendix C. Other Source Searching Algorithms

In this section, we introduce the gradient search algorithm and the uniform search algorithm.

### Appendix C.1. Gradient Search Algorithm

The gradient search algorithm is a light-weighted algorithm that searches for sources by moving along the radiation gradient-rising direction. In this paper, the gradient of radiation was defined as $\frac{d\bar{m}}{dl}$. $d\bar{m}$ was the change of mean radiation measurement, and dl was the change of the measurement position. The gradient search algorithm calculated the radiation gradients of four possible moving directions (up, down, left, right) as $\left(g_{1},g_{2},g_{3},g_{4}\right)$. Then, these gradients were converted to a moving probability vector by the softmax function: $\left(p_{1},p_{2},p_{3},p_{4}\right)=softmax\left(g_{1}/q,g_{2}/q,g_{3}/q,g_{4}/q\right)$. For example, $p_{1}$ represents the probability for the detector to move in the ’up’ direction. *q* is a tunable parameter controlling how much randomness we want to add into the decision process. A large *q* leads to a more evenly distributed random choice over four moving directions, while a small *q* assigns more probability in the moving direction with the largest radiation gradient.

Since the radiation measurements are noisy, the calculated radiation gradients are not always pointing to the correct direction. Thus, it is not always preferred to move along the biggest-gradient direction. Instead, we want to explore other directions. This will help the detector avoid being trapped in local loops. In order to get the best trade-off between moving along the biggest gradient direction and exploring other directions, we conducted two experiments to find the optimal *q* settings for simulation areas without walls (plot(a) of Figure A1) and with walls (plot (b) of Figure A1). For each of the simulation areas, we tried six log-scaled *q* values: {0.03, 0.07, 0.16, 0.35, 0.80, 1.78}. For each *q* value, we repeated the experiment 200 times with fixed source intensity as 1000 cps. As shown in Figure A1, the best *q* was 0.16 for areas without walls, and the best *q* was 0.35 for areas with walls. Those two optimal values were used throughout all other experiments in this paper.

### Appendix C.2. Uniform Search Algorithm

The uniform search algorithm is a traditional source searching strategy that uses a zip-zap pattern to scan the entire searching area [27]. In this paper, we used three uniform search algorithms with low, medium, and high searching densities represented by plot (a), (b), and (c) in Figure A2. The searching density controls the trade-off between searching time and searching coverage.
